# Recent Progress on the Application of Microneedles for In Situ Sampling in Surface-Enhanced Raman Scattering Detection

**DOI:** 10.3390/bios15060350

**Published:** 2025-06-01

**Authors:** Weiqing Yang, Ying Chen, Xingliang Cheng, Shuojiang Liu, Huiqi Zhu, Yuling Hu

**Affiliations:** School of Chemistry, Sun Yat-sen University, Guangzhou 510060, China; yangwq63@mail2.sysu.edu.cn (W.Y.); cheny2268@mail2.sysu.edu.cn (Y.C.); chengxliang@mail2.sysu.edu.cn (X.C.); liushj65@mail2.sysu.edu.cn (S.L.); zhuhq26@mail2.sysu.edu.cn (H.Z.)

**Keywords:** surface-enhanced Raman scattering, microneedle, in situ sampling

## Abstract

The efficient and non-invasive collection of biological samples has become a critical challenge for the continued development of surface-enhanced Raman scattering (SERS). When integrated with minimally invasive microneedle (MN) sampling technology, SERS enhances its applicability in real-time, non-invasive molecular detection. This review focuses on the latest advances in MN-based SERS sensors. Firstly, a comprehensive summary is presented of MN types and research progress in the design and engineering of SERS-active MNs. Then, the sampling method of SERS MNs and the MN-based SERS detection mode are also described in detail. Finally, the applications of SERS MNs in fields such as disease diagnosis, drug monitoring, and food safety are highlighted. Additionally, current challenges are discussed and future development prospects are prospected with the aim of contributing to the design of MN-based SERS sensors for diverse applications.

## 1. Introduction

Surface-enhanced Raman scattering (SERS), as a powerful analytical technique, has the advantages of non-destructive, rapid detection and high sensitivity [1]. It can accurately reflect the fingerprint structural changes in molecules on the surface, which significantly improves the anti-interference ability and achieves the accurate detection of molecules in complex samples [2,3]. This has led to its extensive application in fields such as biomedicine [4], food safety [5], and environmental monitoring [6]. However, despite its significant advantages, SERS still faces some challenges in sample collection and the real-time analysis of samples. The efficient and non-invasive collection of biological samples has emerged as one of the pivotal concerns impinging on the continued evolution of SERS technology.

Microneedle (MN) technology, as an emerging non-invasive sampling technique, has made significant progress in the fields of biological sample collection and drug delivery in recent years [7]. MNs penetrate the superficial layers of skin or biological tissues through tiny needle-like structures, enabling the effective extraction of biological samples or the delivery of drugs without causing significant pain or trauma [8]. In addition, with the progress of material science and manufacturing technology, various types of MNs such as polymer MNs and metal MNs have been widely studied. MNs have been widely used in skin sampling [9,10], drug monitoring [11], drug delivery [12], MN-assisted gene transfer [13], and biosensors [14,15,16]. Significantly, in the context of these applications, the utilization of MN technology for disease diagnosis through the analysis of interstitial skin fluid (ISF) has emerged as the most prominent domain, attracting intense attention from the global research community. ISF is the fluid surrounding skin cells and tissues, serving as a rich and accessible source of biomarkers [17,18]. Therefore, the direct collection of biomarkers from the local skin by MNs can be used for the accurate diagnosis and monitoring of various diseases [19]. MNs are regarded as an ideal tool for ISF analysis [20]. The integrated application of MNs with other technologies is continuously expanding [21]. In particular, the combination of SERS and MN technology to form a sampling and detection system has opened up a new generation of efficient, non-invasive, and sensitive methods for biological sample analysis [22]. The development of the SERS MN system provides a more convenient approach for clinical diagnosis and offers novel solutions for personalized health management, disease monitoring, and food safety [23].

**Figure 1:**
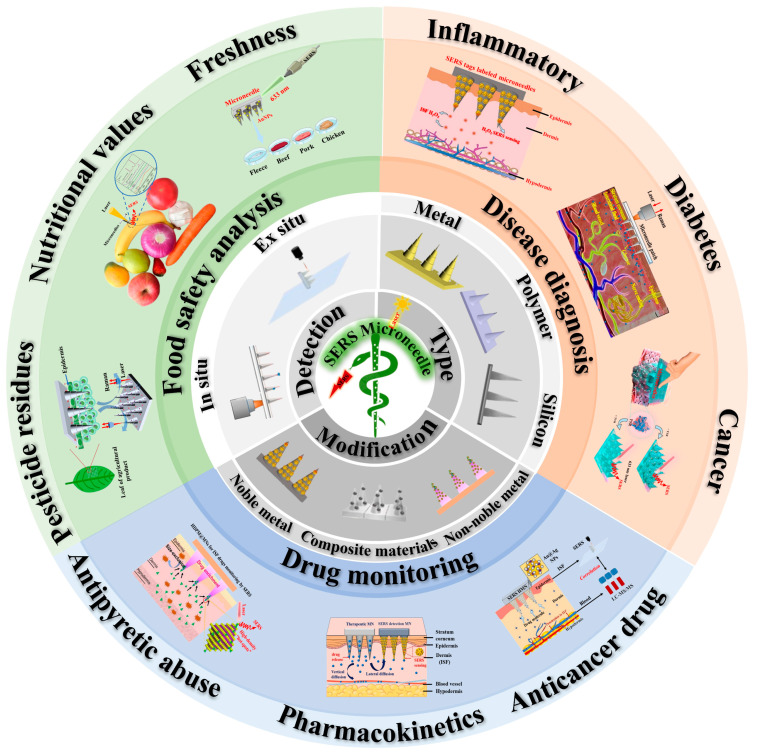
Summary of microneedles for in situ sampling in surface-enhanced Raman scattering detection.

In this review, we comprehensively summarize the latest research progress in the application of MNs for in situ sampling in SERS detection (Figure 1). Firstly, several main types of MNs are introduced, and modifying materials for SERS MN analysis and SERS detection modalities are discussed. Then, the applications of SERS MNs in the fields of disease diagnosis, drug monitoring, and food safety are highlighted. Finally, the challenges and prospects of MNs in SERS analysis are discussed, aiming to maximize their potential.

## 2. Types of Microneedles for SERS Sensing

As a novel non-invasive sample collection tool, MNs have gradually emerged as a crucial carrier for SERS sensors [24,25]. Depending on the design and function, there are various types of MNs, including metal MNs, polymer MNs, silicon MNs, and ceramic MNs. Each type of MN has its unique advantages in terms of structure, material, and shape, which can meet the demands of different application scenarios. Herein, we present several commonly employed MNs for SERS sensing detection and explore their distinctive features as well as potential applications.

### 2.1. Metal Microneedles

Metals such as gold, silver, titanium, and stainless steel are used to fabricate MNs due to their high mechanical strength, excellent ductility, and high electrical conductivity. Laser ablation, laser cutting, metal electroplating, and chemical etching are commonly employed methods for preparing metal MNs [26]. Metals can be utilized to construct both hollow and solid MNs, which empowers metal MNs with broad application prospects in domains such as disease diagnosis, drug monitoring, and food safety [27]. For example, Han et al. utilized MNs made of stainless steel, which were applied for in vivo nitric oxide sampling and subsequent in vitro SERS detection [28]. Kolluru et al. devised steel MNs. This particular MN sensor was composed of a row of nine steel needles, each measuring 650 µm in length. It was capable of extracting ISF from the skin, thereby facilitating further disease diagnosis [29]. Nevertheless, metallic MNs can also induce different levels of risk of inflammatory reactions in biological tissues depending on their different shapes and sizes [30]. For example, a study published by Bal et al. demonstrated that metal MNs of different lengths and geometries induced varying degrees of irritation. Metal MNs with lengths up to 550 μm can be used to overcome the skin barrier function, eliciting the most intense skin irritation among the tested configurations. Overall, MN-induced skin irritation is short-lived, subsiding within less than 2 h. Human volunteers reported their application as painless [31]. Compared to methods such as tape stripping, MNs elicit only minimal and transient irritation. To assess the safety of MNs, Corsini et al. utilized colorimetry and laser desorption/ionization (LDI) to measure irritation potentially induced by metal MNs. Both methods evaluated erythema, which serves as one of the fundamental markers of inflammation. Experimental results demonstrated that MNs with different shapes and lengths elicited varying degrees of erythema, with the skin exhibiting more intense inflammatory responses when MN length exceeded 500 μm [32].

### 2.2. Polymer Microneedles

Polymer MNs have been widely developed due to their good biocompatibility, low cost, ease of molding, and potential for mass production. A wide range of polymer materials, including poly (methyl methacrylate) (PMMA), polydimethylsiloxane (PDMS), polystyrene (PS), polylactic acid (PLA), polycarbonate (PC), polyethylene (PE), polyurethane (PU), polyether ether ketone (PEEK), Norland Optical Adhesive (NOA), and cyclic olefin polymers, have been utilized for MN manufacturing [33]. Polymers are typically very tough, allowing MNs to avoid brittle fracture during insertion into the skin or other tissues. Since most polymers are biocompatible, polymer MNs rarely cause severe side effects. Moreover, some polymers are biodegradable or water-soluble, enabling drug encapsulation in dissolvable MNs. Upon insertion, the drugs are released as these MNs degrade or dissolve. This eliminates the need for disposing of sharp, biohazardous waste.

Recently, MNs have been widely applied in the construction of SERS sensors. For example, Chen et al. developed a flexible plasma PMMA MN array-based SERS sensor for the convenient and sensitive monitoring of pH in skin ISF [34]. Mei et al. developed MNs made of PMMA for the continuous detection of ISF using MN patches for the dynamic tracking of acute peritonitis [35]. He et al. used the NOA61 light-curing adhesive to prepare transparent MNs and successfully constructed SERS MNs for in situ analysis for the tracking and monitoring of mouse melanoma [36]. Li et al. prepared SERS MNs made of a mixture of PU and sericin protein (SF) with sufficient mechanical strength for skin penetration and high light transmission for SERS signal detection [37]. Vitoria et al. prepared MNs made of PDMS for the SERS detection of synthetic drugs in plasma [38]. However, polymer MNs also face some challenges. A major issue is that some of the polymers are soft, which may lead to catastrophic buckling during insertion [39]. This is especially problematic in blood sampling, where the MNs are longer than those used for drug delivery, making buckling more likely [40,41]. Regarding the biocompatibility and toxicity of polymeric MNs, Hoesly et al. reported that upon the insertion of polymeric MNs into the skin, the reaction was characterized by spontaneous, nearly imperceptible erythema that resolved rapidly without external intervention [42]. Similarly, the polymeric MNs used in a study by Rouphael et al. were well tolerated, eliciting only mild pruritus, tenderness, and erythema [43]. Haq et al. demonstrated that the skin disruption caused by polymeric MNs was transient, with self-repair occurring within 24 h. They further showed that the channels created by MNs were smaller and healed faster than those from subcutaneous injection needles, thereby reducing the risk of pathogen exposure [44].

### 2.3. Other Microneedles

In the early stages of MN development, silicon was used as a material for fabricating MNs. Despite silicon’s high fracture strength and toughness, the brittleness of silicon still poses a risk that the sharp tips of silicon MNs may fracture under the skin during application [34]. The experiments by Henry et al. showed that some silicon MNs may break off near the tip (top 5–10 μm) after insertion into the skin [45]. Additionally, silicon is not an FDA-approved biomaterial, and its biocompatibility has not been well established to date. Therefore, these fractured silicon fragments left under the skin may lead to undesired immunogenic consequences or may be transported via blood vessels to the heart, causing arterial blockage [46]. Like silicon MNs, glass MNs also suffer from brittleness issues during application. Matthaei and colleagues used hand-pulled, 25° beveled glass MNs to deliver ink into the eyes of mice. Their results showed that the glass needles tended to provide the smallest absolute affected area, while injections using Hamilton 33G and WPI 35G needles showed a trend in larger absolute stained areas, though the glass needles could fracture within the corneal stroma. Experiments have demonstrated that borosilicate glass implants appear biocompatible with the cortical surface [47,48], yet silica glass carries a risk of inducing granulomas in the skin [23]. For pharmaceutical applications, borosilicate glass may be preferable to silica glass.

Ceramic materials have attracted significant interest in MN fabrication due to their superior properties, such as high strength, excellent chemical resistance, and biocompatibility [49,50]. Notably, the surface area, porosity, and degradation of ceramics can be tailored during manufacturing, enabling the design of ceramic MNs for controlled drug release [51]. For example, Boks et al. fabricated alumina-based nanoporous ceramic MNs for the controlled release of model vaccines [52]. It is important to note that while studies indicate a need to consider the risk of aluminum release from alumina MNs during long-term skin insertion, short-term applications have demonstrated good biocompatibility with minimal adverse reactions such as redness or swelling [53]. Cai et al. [54] developed solidified bioceramic MNs for transdermal drug delivery, showing that their porosity and resorbability can modulate release behavior while maintaining sufficient mechanical strength to penetrate skin without fracture. Ovsianikov et al. successfully prepared hollow microneedles using a two-photon polymerization of Ormocer^®^ US-S4 (organically modified ceramic; Fraunhofer-Gescllschaft, Munich, Germany), an amorphous organic–inorganic hybrid material. Their research demonstrated that their Ormocer^®^-based MNs were strong enough to penetrate porcine skin without breaking and exhibited minimal impact on the growth of human epidermal keratinocytes [55]. Researchers have explored several types of inorganic and carbon materials as MN sensors. For example, Kang et al. modified dendritic gold (Au-CFNs) on MNs made of carbon fiber, resulting in the preparation of MNs with SERS activity, which were used to detect acetamiprid [56].

## 3. Modification of Microneedles for SERS Sensing

Since MNs themselves cannot generate SERS signals, it is necessary to modify the MNs with materials that can induce SERS activity, such as noble metal nanomaterials and some non-noble metal nanomaterials. These nanomaterials can induce significant localized surface plasmon resonance (LSPR) under electromagnetic radiation. This greatly enhances the Raman signals of target molecules, enabling ultra-high SERS sensitivity [57,58]. The nanomaterials used for modifying SERS MNs mainly include gold, silver, copper, metal oxides, metal–organic frameworks (MOFs), conjugated polymers, two-dimensional materials, and their composite materials.

### 3.1. AuNP-Based Modification

**Figure 2:**
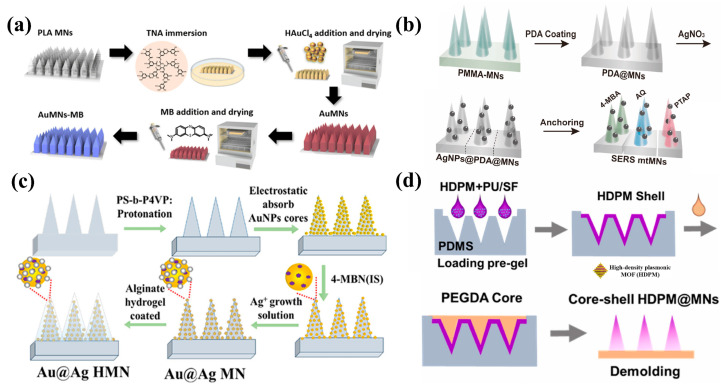
(**a**) Preparation process of microneedles modified with AuNPs. Reprinted with permission from [70]. Copyright 2023 Royal Society of Chemistry. (**b**) Preparation process of microneedles modified with AgNPs. Reprinted with permission from [71]. Copyright 2020 Elsevier B.V. (**c**) Preparation process of microneedles modified with Au@Ag NPs. Reprinted with permission from [72]. Copyright 2024 American Chemical Society. (**d**) Preparation process of microneedles modified with high-density plasma MOFs. Reprinted with permission from [37]. Copyright 2024 Elsevier B.V.

Gold nanoparticles (AuNPs) are widely utilized as SERS substrates due to their excellent optical properties, favorable chemical stability, ease of preparation, and amenability to functionalization [59]. AuNPs exhibit excellent chemical stability and low biological toxicity, making them a preferable choice under most complex conditions, particularly in biological applications [60]. The core of the SERS enhancement mechanism of Au arises from the localized surface plasmon resonance (LSPR) effect: when light irradiates the surface of AuNPs, free electrons collectively oscillate to form plasmon waves, generating intense localized electromagnetic fields at the nanostructure surface. The particle size and morphology of gold nanoparticles directly influence the position and intensity of LSPR peaks. Various shapes of AuNPs, such as spherical [61], triangular [62], rod-like [63], cubic [64], star-shaped [65], flower-like [66], etc., have been modified onto MNs to endow them with SERS activity. The sharp structures of these shapes generate “hotspot” regions with extremely high electromagnetic fields. When the spacing between AuNPs is less than 10 nm, the LSPR of adjacent particles couples to form a stronger electromagnetic field, known as the “gap enhancement” effect [67]. For example, Vo Thi et al. investigated the fabrication of gold nanoflower structures on MNs for use as SERS sensors for intradermal sensing applications [68]. Wang et al. investigated the use of MN patches treated with ozone and coated with gold nanopopcorn (GNpops) with SERS activity for the detection of Escherichia coli [69]. He et al. used photocrosslinked NOA61 to prepare sulfhydryl microneedles, which could be used to anchor AuNPs, and successfully constructed SERS MNs for in situ detection [36]. Chia et al. developed an AuNP deposited on transparent PLA MNs for SERS sensing and antimicrobial photodynamic therapy (Figure 2a) [70].

### 3.2. AgNP-Based Modification

When compared to AuNPs, silver nanoparticles (AgNPs) exhibit lower stability, yet their lower imaginary part of the dielectric constant in the visible and near-infrared regions tends to generate stronger localized electromagnetic fields and SERS enhancement, endowing them with enormous application potential in the SERS field [73]. Analogous to the SERS enhancement effect of AuNPs, the enhancement mechanism of AgNPs primarily originates from the synergistic effect of LSPR and charge transfer (CT) mechanisms, with the enhancement efficiency also dependent on size, morphology, interparticle spacing, and aggregation state [74]. By combining AgNPs with MNs, a SERS sensor based on MNs is developed for the detection of analytes. For example, Ju et al. made a non-swelling hydrogel MN using PMMA coated with 1-decanol (to bind to glucose) and AgNPs (to enhance the Raman signal) [75]. Peng et al. developed SERS MNs (AgNPs@PDA@MNs) modified with polydopamine and decorated with AgNPs as a multiplexed SERS diagnostic platform, demonstrating a remarkable ability to detect complex inflammation in vivo, in situ, and in real time (Figure 2b) [71]. Gu et al. used 4-mercaptophenylboric acid (4-MPBA)-modified AgNPs as SERS probes for the detection of the tyrosinase (TYR) concentration in the skin [76]. Yuen et al. presented a Ag-coated MN for detecting low concentrations of glucose analytes located on the surface of a skin model at a depth of more than 700 μm for simulating intradermal SERS measurements [77].

### 3.3. Composite Metal-Based Modification

In addition to modifying MNs with materials such as AuNPs or AgNPs, composite metal materials can also be synthesized by doping other noble metals like platinum or palladium, which can provide a more effective enhancement of the SERS signal. By combining different metals and leveraging the overlap or complementarity of their LSPRs, the spectral response range can be expanded and electromagnetic fields enhanced [78]. Electron redistribution at the heterostructure interface strengthens the interaction between molecules and materials. For example, in Au@Ag core–shell particles, the LSPR of the Ag shell couples with the plasmon resonance of the Au core, generating a broadband enhancement in the visible-to-near-infrared region [79]. Li et al., using a novel nucleo-satellite structure of an Au@Ag SERS substrate with hydrogel wrapped around the MNs, achieved sensitive and quantitative drug detection in mouse ISF within 10 min (Figure 2c) [72]. Huang et al. improved the analytical reliability and sensitivity by modifying trimetallic Au@Ag-Pt NPs on MN patches for SERS and the colorimetric dual-mode detection of TYR in human skin [80]. Vinu Mohan et al. used the minimally invasive detection of subcutaneous alcohol by means of a sensing device with a pyramid-shaped MN structure integrated with Pt and Ag [81]. Although noble metal materials offer better SERS enhancement effects, they are toxic and can compromise the biocompatibility of SERS MNs. Therefore, it is particularly important to explore low-cost, non-toxic, and highly biocompatible MN modification materials with good enrichment effects for SERS MN sensing technology. Such materials would enable the development of safer and more effective SERS MNs, while maintaining both enhanced performance and biological compatibility.

### 3.4. MOF-Based Modification

Non-noble metal materials, due to their good biocompatibility, low cost, and excellent enrichment performance, have gained significant attention [82,83]. Among them, as a class of novel hybrid organic–inorganic supramolecular materials, MOFs possess large surface areas, porosity, chemical stability, and uniform tunable nanostructured cavities, offering enormous potential for the material modification of SERS-active MNs [84]. MOF materials are typically regarded as templates for loading densely packed and well-dispersed SERS-active nanoparticles, with the SERS enhancement effect attributed to the role of noble metal particles [85,86]. When MOFs are combined with metal nanoparticles, the confinement effect of MOFs integrates with the LSPR effect of metals, enhancing both their molecular adsorption capacity and electromagnetic fields (e.g., Au particles form localized hotspots within MOF pores) [87,88]. Sun et al. [89] achieved the transformation of MOF materials from non-SERS-active to SERS-active substrates by fine-tuning their metal centers, organic ligands, and framework topologies, obtaining an enhancement factor as high as 10^6^. MOF materials have been used in the field of the analytical detection of SERS MNs. For example, Li et al. designed a SERS MN sensor utilizing high-density plasma MOFs (HDPMs) for the real-time non-destructive monitoring of acetaminophen concentration in ISFs with a quantitative detection window of 1~100 μM and a lowest detection limit of 0.45 μM (Figure 2d) [37].

### 3.5. MXene-Based Modifications

MXenes are transition metal carbides/nitrides that are highly conductive semi-metallic materials capable of generating plasma resonances in the visible or near-infrared range [90]. MXenes, with their metallic conductive surfaces, can support surface plasmon oscillations to generate electromagnetic field enhancement. Functional groups on MXene surfaces can form hydrogen bonds or electrostatic interactions with molecules, promoting charge transfer [91]. By modifying the surface or intercalating ions, the Fermi level of MXenes can be tuned to optimize charge transfer efficiency, thereby enhancing SERS signals. The two-dimensional structure provides a large specific surface area and abundant surface active sites, enabling high molecular adsorption; meanwhile, surface delocalized electrons can strongly interact with molecules to improve Raman scattering efficiency [92]. When MXenes are loaded with Au/Ag nanoparticles, the LSPR of metals synergizes with the conductive surfaces of MXenes to form a dual enhancement of “hotspots” and a high adsorption efficiency. Sarycheva et al. first demonstrated that 2D Ti_3_C_2_T_x_ MXene exhibits good SERS activity as a substrate, with an enhancement factor (EF) up to 10^6^. An important potential advantage of MXenes is their ability to deposit materials on different substrates, including flexible ones [93]. Soundiraraju et al. prepared different SERS substrates by loading Ti_2_NT_x_ on paper, silicon, and glass. Their results showed that Ti_2_NT_x_ on paper-based substrates had the highest enhancement efficiency, reaching up to 10^12^ [94]. One key advantage of MXenes is their potential as flexible materials when combined with MNs. Soundiraraju et al. prepared various SERS substrates by loading paper, silicon, and glass with Ti_2_NT_x_. The results showed that Ti_2_NT_x_ provided the highest enhancement efficiency on paper-based substrates, achieving an enhancement factor of up to 10^12^ [94]. Additionally, Yang et al. incorporated the ultra-flexible property of Ti_2_NT_x_ MXene into a SERS microfluidic chip, significantly improving the detection sensitivity of SERS microfluidic sensors [95]. While the development of MXene-based substrates is still in its early stages, the reported results suggest that MXene materials hold great promise for use in wearable, real-time monitoring sensors in combination with MN patches for skin applications.

## 4. Microneedle Sampling for SERS Detection

### 4.1. Microneedle Sampling Mode

**Figure 3:**
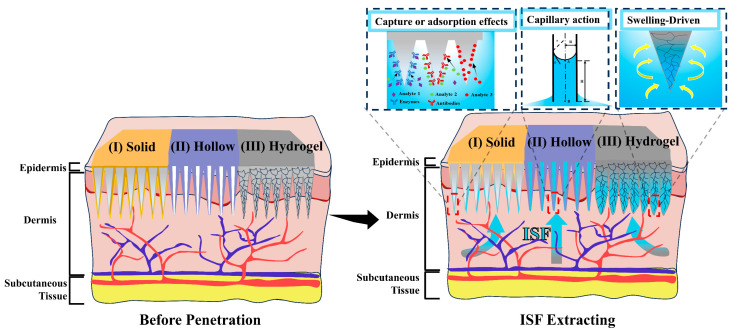
Microneedle architectures for extraction of biological sample. Representation of solid, hollow hydrogel microneedles.

MNs can be used as diagnostic tools for extracting analytes from body fluids (e.g., ISF, blood, sweat, etc.) and plant tissue fluids. Overall, current MN-based diagnostic platforms use the following methods for the collection of biological samples (Figure 3) [96,97]: (I) solid MNs for the direct physical adsorption of analytes; solid MNs coated with specific enzymes, antibodies, or other molecules to capture the target analytes of interest [98,99]; (II) hollow MNs utilizing capillary forces to extract biomolecules; and (III) hydrogel MNs exploiting their swelling properties to extract analytes. After multiple insertions of solid MNs, micropores are formed in the biological epidermis, allowing biological fluids to reach the surface through these micropores, where the target biological samples are adsorbed onto the MN surface. The body of hollow MNs used for biological sample extraction consists of numerous interconnected pores that enable capillary forces to extract biological samples. The hydrogel MNs swell upon insertion into the biological epidermis due to the diffusion gradient and absorb biological samples [100,101]. When MNs are connected to an integrated SERS biosensor to detect biological samples, they can function as biosensing probes [102].

#### 4.1.1. Solid Microneedles

**Figure 4:**
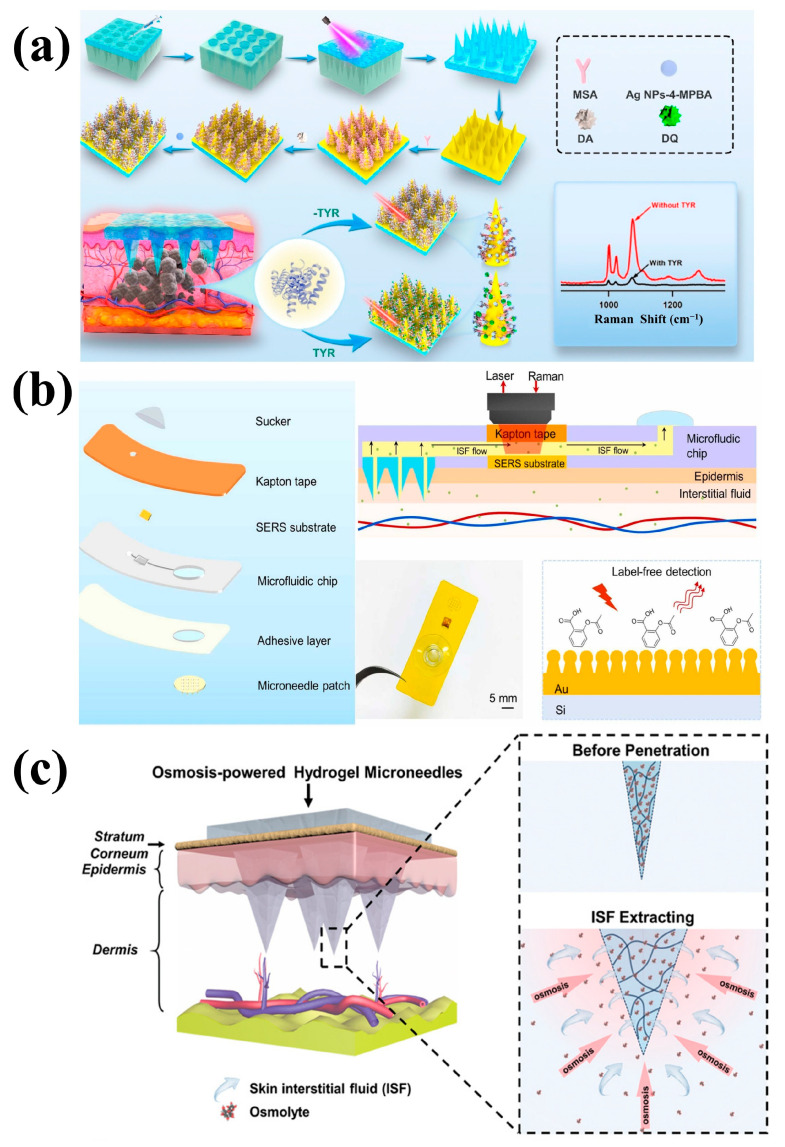
(**a**) The principle of SERS solid microneedle biosensors for the in situ detection of TYR. Reprinted with permission from [76]. Copyright 2024 Elsevier B.V. (**b**) Schematic diagram of wearable sensors for sampling and detection using SERS hollow microneedles. Reprinted with permission from [113]. Copyright 2024 Elsevier B.V. (**c**) Schematic diagram of skin ISF extraction using hydrogel microneedles. Reprinted with permission from [114]. Copyright 2020 John Wiley and Sons Ltd.

The surface of solid MNs can capture target substances of interest, such as proteins, nucleic acids, pathogens, or small molecules, by physical adsorption or chemical modification [103,104]. For example, Mukerjee et al. pioneered the use of micromachining techniques to extract ISF using 250–350 µm long solid MNs made of monocrystalline silicon [105]. Li et al. coated a hydrogel layer on SERS solid MNs to form SERS HMNs, which combined the advantages of both sampling MNs and detection MNs. The hydrogel layer not only facilitated the rapid extraction of ISF and promoted the adsorption of drug molecules onto the SERS MN substrate but also prevented the Au@Ag nanoparticles from detaching during the skin insertion process, significantly improving detection performance [72]. To enhance the specificity of sampling and detection, the surface of the solid MNs can be modified with specific enzymes, antibodies, or other molecules. These biomolecules can specifically bind to the target substances, thereby improving capture efficiency. For example, modifying the surface of solid MNs with specific antibodies enables them to selectively recognize and capture a particular antigen or pathogen, while enzyme modifications can react with target substrates to generate detectable signals [106]. For example, Gu et al. introduced a new sensor platform that integrates solid MN biosensors with SERS technology for the in situ detection of TYR in human skin. The platform modifies solid MNs with mercaptoacetic acid to capture dopamine through an amide reaction between functional carboxyl (-COOH) and amine (-NH_2_) groups. AgNPs modified with 4-MPBA serve as the SERS probes, forming a stable cyclic boronic ester through the interaction between the boronic acid group of 4-MPBA and hydroxyl groups. When the MNs penetrate the skin containing TYR, dopamine is oxidized to dopamine quinone, disrupting the interaction between AgNP-4-MPBA and the MN array, leading to a decrease in the SERS signal (Figure 4a). The intensity of the SERS signal is inversely proportional to the increase in TYR concentration, allowing for the detection of TYR levels [76]. Takeuchi et al. fabricated porous PDMS MNs coated with hyaluronic acid (HA) to aspirate glucose from ISF via the mechanical compression of the PDMS matrix. By optimizing porosity, they created MNs (60% porosity) that successfully penetrated skin and extracted ISF in vivo at a rate of 0.46 μL/min. Glucose extraction was confirmed by color changes in glucose test strips placed on the MN backsides [107]. Al Sulaiman et al. proposed poly-l-lactide MN patches coated with an alginate–peptide nucleic acid hybrid material for the multiplex sampling of specific cancer-related microRNAs from ISF. The MNs sampled up to 6.5 μL of fluid within 2 min at a rate of 0.74 μL/min, outperforming existing sampling techniques. They also showed high specificity and sensitivity in human skin biopsies, detecting targets as low as 6 nM [108]. Zhang et al. created MNs from a mixture of poly(ethylene glycol) diacrylate and poly(ethylene glycol), integrated with photonic crystal barcodes for the non-invasive detection of ISF inflammatory cytokines. Photonic barcodes modified with specific probes enabled biomarker enrichment and detection via immunofluorescence in a sepsis mouse model. These encoded MNs outperformed existing MNs in ISF detection, featuring simplified procedures, a multiplexing capability, and the in vivo detection of targets with retained biological activity [109]. Wang et al. introduced polystyrene MN patches functionalized with biorecognition elements to selectively capture protein biomarkers. The patches exhibited sufficient mechanical strength to penetrate skin without bending, with minimal invasiveness and excellent biocompatibility. When combined with a plasmonic fluorescence-linked immunosorbent assay (p-FLISA), they enabled sensitive local biomarker detection in mice [110]. Simas et al. modified PDMS MNs with ligand-coated gold nanoparticles to detect potent abused drugs in ISF. Combined with SERS and mass spectrometry, this multimodal detection method distinguished fentanyl, alprazolam, or their mixtures in human patient samples with high accuracy [111]. Wu et al. conjugated functionalized trimethylolpropane ethoxylate triacrylate MNs with antibodies to detect anemia biomarkers. Using immunofluorescence and aptamer-based fluorescence quenching, the MNs showed high specificity and sensitivity, generating results within 20 min. Sensitivity for ferritin, folic acid, and vitamin B12 was significantly enhanced compared to traditional methods, achieving lower LODs. The integrated device maintained sensitivity for up to 21 days without obvious degradation, indicating practical usability and stability [112].

#### 4.1.2. Hollow Microneedles

Each needle of the hollow MN array has a small aperture, allowing it to effectively penetrate the biological surface without damaging deeper tissues, making it safe and painless. Through these porous structures, body fluids can be drawn into the MN using capillary action or negative pressure, and then collected for biological sample detection and analysis [115]. For example, Wang et al. used hollow glass MNs, ranging from 700 to 1500 µm in length, to penetrate the skin. ISF was extracted from the introduced pores using vacuum pressure, with 1–10 µL of ISF collected for glucose measurements within 2–10 min [116]. Miller et al. reported a series of hollow MNs, which extracted 20 µL and 60 µL of dermal ISF from human and rat subjects, respectively. The MN array, with a length of 1500 μm for each needle, extracted up to 16 µL of ISF from human subjects within 2 h [117]. Xiao developed a microfluidic-based MN biosensor for portable ISF extraction and ultra-sensitive uric acid monitoring. The device consisted of a hollow MN patch and a flexible microfluidic chip as the sampling part, with SERS as the sensing part. Under the action of capillary forces and suction-based negative pressure, the ISF was transported through microchannels to the sensing interface for further uric acid analysis (Figure 4b). The well-designed SERS MN biosensor was integrated with a handheld Raman spectrometer, contributing to precise and personalized daily health monitoring [113].

#### 4.1.3. Hydrogel Microneedles

Hydrogel MNs are MN arrays made from hydrogel materials that possess strong swelling capabilities. Hydrogel materials typically have excellent biocompatibility, making them less likely to cause allergic reactions, and thus suitable for contact with human skin. When hydrogel MNs come into contact with the skin, the hydrogel absorbs moisture from the skin and expands, creating tiny pores that help effectively collect body fluids from the skin’s surface or deeper layers [103,118]. Through this swelling process, hydrogel MNs enable non-invasive, rapid fluid sampling with minimal skin damage, and the process is nearly painless. For example, Zheng et al. introduced an osmotic-driven hydrogel MN patch that extracts ISF three times faster than existing platforms and provides an in situ analysis of the extracted biomarkers. During the extraction process, the osmotic agent dissolves in the matrix and generates osmotic pressure, enhancing the diffusion of ISF from the skin into the hydrogel matrix (Figure 4c). The patch, with 100 MNs, can extract 7.90 μL of ISF from ex vivo pig skin and 3.82 μL of ISF from in vivo mouse skin within 3 min [114]. In addition to fluid sampling, hydrogel MNs can also be used as a drug delivery system, directly delivering medication to the deeper layers of the skin to enhance its therapeutic effects. Fan et al. proposed a novel strategy for achieving multifunctional and controlled drug delivery by integrating light-responsive drug-delivery microspheres into a MN array. The composite MN system possesses sufficient mechanical strength to penetrate the skin and deliver drugs uniformly beneath the skin. It has been shown that the composite MN system can serve as an excellent drug delivery system with significant practical value in clinical medicine [119]. Zhou et al. used fabricated hydrogel MNs for the transdermal delivery of bovine serum albumin (BSA) and experimentally demonstrated that these hydrogel MNs exhibited no cytotoxicity. Moreover, they showed triple sensitivity to pH, temperature, and glucose levels, enabling more precise on-demand drug delivery [120].

### 4.2. SERS Detection Mode

The detection mode of SERS MNs varies according to the material of the MN itself and is mainly categorized into indirect SERS detection and in situ SERS detection [121]. If the MN is made of transparent polymers, such as NOA [122], PMMA [75], etc., it is possible to detect the target molecule by using the MN as an in situ SERS test. If the MN is made of an opaque material, e.g., metallic MN, ceramic MN, etc., this type of MN cannot be penetrated by the laser and the SERS signal of the target molecule can only be detected by indirect methods. Indirect detection involves extracting biological fluids using the MN, followed by an in vitro analysis of the extract to identify analytes through binding to a SERS substrate [123].

#### 4.2.1. In Situ Detection

**Figure 5:**
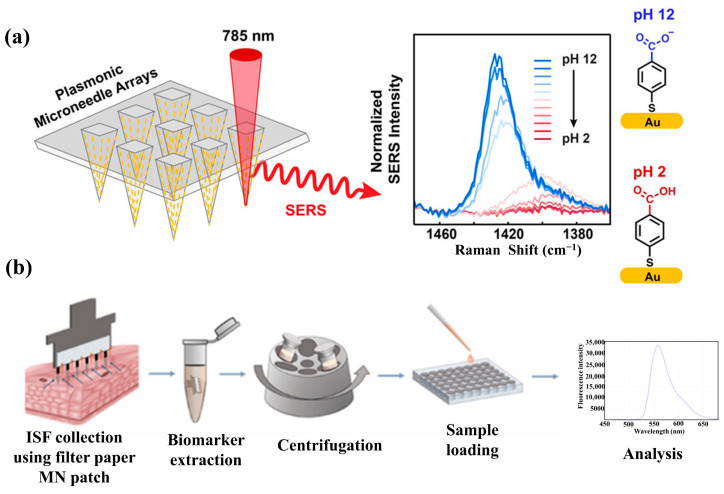
(**a**) Schematic diagram of plasma microneedle array for SERS in situ detection. Reprinted with permission from [122]. Copyright 2019 American Chemical Society. (**b**) Schematic diagram of plasmonic paper microneedle patch for SERS ex situ detection. Reprinted with permission from [29]. Copyright 2019 American Chemical Society.

SERS MN in situ detection technology can obtain the required detection data in situ, in real time, and with high accuracy, greatly improving detection efficiency and reliability. Ju et al. reported a novel SERS sensor based on a low-cost PMMA MN array for in situ skin glucose detection. After incorporating 1-dodecylthiol onto the surface of the silver-coated array, the sensor was calibrated in a skin model within the 0–20 mM glucose range, and then tested for in vivo glucose quantification in a streptozotocin-induced type 1 diabetic mouse model. The results showed that the functional PMMA MN array (F-PMMA MN) could directly measure glucose in ISF within minutes, maintaining its structural integrity without swelling. The high transmittance of PMMA allowed laser and Raman signals to pass through the MNs, enabling the in situ monitoring of blood glucose levels in diabetic patients. This polymer-based MN array SERS biosensor holds potential for painless glucose monitoring in diabetic patients in the future [75]. Park et al. reported the integration of SERS with a MN array as a minimally invasive chemical sensing platform, particularly for sensing ISF. The MN array is made of commercial polymer adhesives and coated with plasma-active gold nanorods, which are functionalized with pH-sensitive molecules 4-mercaptobenzoic acid. This sensor can quantify pH values within the range of 5 to 9 and can detect pH levels in agarose gel skin models and human skin in situ. This work is the first to integrate SERS-active nanoparticles with polymer MN arrays and demonstrates the method for in situ sensing using this platform (Figure 5a) [122,123]. Xiao et al. reported a microfluid-based wearable plasmonic MN sensor for ISF sampling and minimally invasive uric acid monitoring. Under the negative pressure generated by finger movement, the hollow MN array extracts subcutaneous fluid through microfluidic channels and delivers it to the sensing chamber for SERS detection. The SERS sensing chip based on a three-dimensional (3D) gold nanorod array was found to exhibit good reproducibility with a 5.96% RSD. This high-density microarray demonstrated the ultrasensitive and label-free detection of uric acid molecules, with a detection limit of 0.51 µM. Integrated with a handheld Raman spectrometer, it allows the rapid in situ identification of target molecules. The sensing platform holds great potential for efficient healthcare and early disease prevention in daily life [113].

#### 4.2.2. Ex Situ Detection

In contrast to the direct in situ detection of SERS MNs, most analyses through MN sampling are realized through ex situ detection. Yi et al. proposed a novel MN patch-based SERS sensor. The tips and bottoms of the MN patch can simultaneously detect pesticide residues on the surface and inside agricultural products. The AgNPs and sodium hyaluronate/polyvinyl alcohol (HA/PVA) hydrogel used in the sensor effectively amplify the Raman signals of pesticide residues. Additionally, the HA/PVA hydrogel can efficiently and rapidly collect residues, making the sensor more convenient for detecting pesticide residues. The stepped structure of the MN increases the surface area of the sensor. This sensor can simultaneously detect pesticide residues of carbendazim and thiophanate-methyl, with detection limits of 10^−7^ M and 10^−8^ M, respectively. The detection process is minimally invasive and harmless to agricultural products. The application of this MN patch-based SERS sensor can be extended to the safety and health monitoring of other plants and animals [5]. Kolluru et al. developed a SERS MN patch for the analysis of biomarkers in ISF. MNs create micropores on the skin surface, allowing micro-liter amounts of ISF to be collected onto plasma paper on the back of the patch through the micropores. Plasma paper is prepared by immobilizing gold nanorods (AuNRs) coated with poly(styrenesulfonate) (PSS) onto thin filter paper strips. The negatively charged PSS is used to bind the positively charged model compound, Rhodamine 6G (R6G), thereby positioning the R6G on the surface of the AuNRs. The R6G bound to the AuNR surface is detected and quantified by obtaining SERS spectra from the plasma paper MN patch. This method is used to determine the in vivo pharmacokinetic characteristics of R6G in rat ISF and serum (Figure 5b). Their study demonstrates that their plasma paper MN patch has the potential to enable the measurement of molecules in ISF on the patch for research and future medical applications [29]. Mei et al. achieved the patch sensing of H_2_O_2_ biomarkers via the SERS MN technique, whereby a MN patch was inserted into the abdominal skin of mice, and after 15 min, the MN patch was removed from the skin and detected ex situ for SERS signals for the dynamic tracking of acute peritonitis [35]. Huang et al. reported a sensing platform that combines a wearable MN patch and trimetallic Au@Ag-Pt nanoparticles for dual-mode SERS and the colorimetric detection of in situ TYR on human skin for potential melanoma screening. In the presence of TYR, catechol fixed on the MN is preferentially oxidized to quinone, competitively hindering the interaction between the MN and Au@Ag-Pt NPs, triggering the conversion between SERS and colorimetric signals. Using the B16 F10 mouse melanoma model, this platform can non-invasively pierce the skin surface and detect TYR levels before and during anti-PD-1 antibody treatment. The platform offers an effective approach for the early prediction, monitoring, and prognosis of skin melanoma [80].

### 4.3. Accuracy and Reproducibility of MN-SERS System

#### 4.3.1. Consistency and Accuracy of ISF Sampling Using Microneedles

To achieve consistent and accurate ISF collection using MNs under diverse skin conditions, improvements are required in MN design, material optimization, sampling protocols, and auxiliary technologies. For varying skin thicknesses, design MNs with a hardness gradient from base to tip (e.g., rigid silicon/metal base with a flexible hydrogel tip). The rigid portion penetrates the stratum corneum, while the flexible tip adapts to different thicknesses, preventing vascular damage from over-penetration [57]. For fluctuating humidity or temperature, employ shape memory alloys (e.g., nitinol) or thermoresponsive polymers (e.g., poly(N-isopropylacrylamide)) to trigger length changes in response to environmental cues, ensuring consistent penetration depth [58]. For sensitive skin, apply ultrasonic or micromachined vibration devices to enhance skin microcirculation and ISF exudation. Alternatively, integrate micropumps into MN patches with pressure sensors to monitor ISF filling status in real time [59]. For pathological skin conditions, administer trace isotopic markers (e.g., ^13^C-glucose) prior to sampling to serve as internal standards for calibrating volume fluctuations. This approach has been shown to reduce the coefficient of variation (CV) for glucose detection in diabetic patients from 14% to 5.2% [60]. For different age groups, leverage physiological signals (e.g., heart rate variability, skin conductance) collected by wearable devices (e.g., smartwatches) to assess skin microcirculation via AI models. Dynamically adjust sampling parameters (e.g., extending sampling time or increasing stimulation intensity) when reduced blood flow is detected [46].

#### 4.3.2. Reproducibility of SERS Detection

Indeed, the sampling accuracy not only depends on sample collection efficacy through the MN system but also on the mechanism enhancement of the biomolecules’ Raman signals through metallic nanoparticles. Multiple factors influence the reproducibility of SERS signals in MN-SERS systems, including instrumental conditions, the selection of MN materials and structures, the uniformity of nanostructures, and the stability of MNs and nanostructures, as well as shelf life. Researchers enhance the reproducibility of SERS signals in MN-SERS systems by synthesizing composite materials, designing functionalized materials, developing MN materials, and performing functional modifications on MNs. For example, Ju et al. leveraged the high light transmittance and mechanical strength of PMMA-MNs to focus laser beams into the dermis for Raman testing, enabling the acquisition of rich physiological information. This approach avoids losses caused by extracting fluids from the skin using MNs and then removing them for Raman detection, thereby improving SERS signal reproducibility [75]. Peng et al. coated biocompatible polydopamine on PMMA-MNs to strongly bind AgNPs, followed by the assembly of 4-MBA, AQ, and PATP molecules, demonstrating good stability and repeatability. The developed MN-SERS sensing system enabled the in situ and real-time acquisition of pH, redox potential, and ROS multiplex information for inflammation diagnosis [71]. Li et al. prepared MN sensors integrated with high-density plasmonic MOFs. Their unique large specific surface area, specific pore structure, and high-density gold nanosphere-filled structure endowed the sensors with efficient ISF enrichment and excellent SERS performance. Calculations show that the maximum electric field enhancement factor of this MN sensor structure reached 5.73 × 10^7^, exhibiting outstanding stability and reproducibility [37]. In microscale SERS detection, the measured SERS signal significantly weakens when the laser spot deviates from the hotspot region of the sample. To improve the reproducibility of SERS signals in MN-SERS system detection, the laser spot should cover the hotspot region of the MN tip as much as possible. Although the structure of MN-SERS substrates remains relatively stable during storage, it may still be affected by contamination or deterioration. To prevent these issues, vacuum protection is typically employed to store the substrates in a dry and clean environment, avoiding contact with potentially contaminating substances to ensure the stability and reproducibility of their performance.

## 5. Application of SERS Microneedle

**Figure 6:**
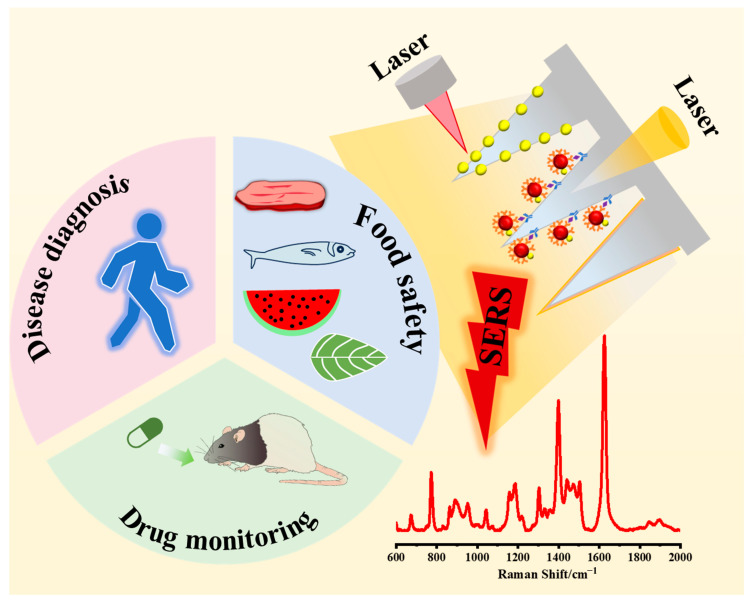
Schematic diagram of SERS microneedle application.

It has been reported that with the integration of MNs with SERS technology, the use of MNs has expanded to biomedical disciplines, including the long-term management of diseases and conditions, therapeutic diagnostics, localized drug delivery, immunobiological management, disease assessment and diagnosis, and cosmetic skincare products. Moreover, with the advancement of technology, SERS MNs are now applied not only in the biomedical, disease diagnosis, and drug delivery fields but also in food safety and other areas. This chapter reviews the applications of SERS MNs in biomedicine, disease diagnosis, and food safety in the last five years (Figure 6). Table 1 comprehensively summarizes the applications of MNs in SERS analysis, including MN types, modification materials, detection methods, analytical applications, and analytical performance.

### 5.1. Disease Diagnosis

**Figure 7:**
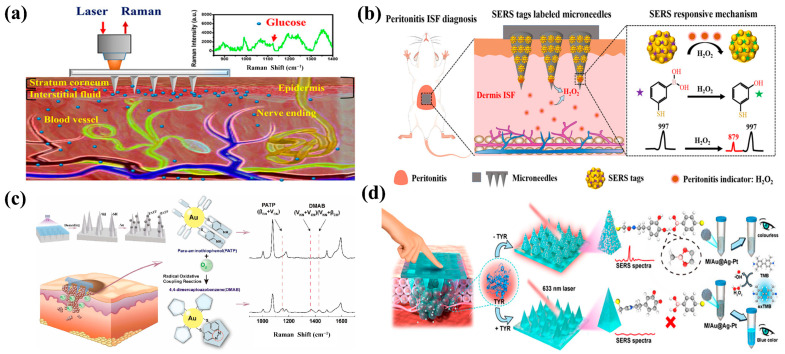
(**a**) Schematic diagram of the glucose measurement using a microneedle array for in vivo transdermal detection based on SERS. Reprinted with permission from [75]. Copyright 2020 American Chemical Society. (**b**) Schematic diagram of evaluating peritonitis development via the skin ISF route. H_2_O_2_ produced in abdomen skin ISF is served as a peritonitis indicator, which can be dynamically detected by minimally invasive and painless SERS tag labeled microneedles. Reprinted with permission from [35]. Copyright 2023 American Chemical Society. (**c**) Schematic diagram of the ultrasensitive detection of superoxide anion radical mutations in melanoma mice with SERS microneedles. Reprinted with permission from [36]. Copyright 2024 Elsevier B.V. (**d**) Schematic diagram of a wearable microneedle patch for the in situ monitoring of tyrosinase for clinical melanoma screening. Reprinted with permission from [80]. Copyright 2023 American Chemical Society.

SERS MNs have been used as disease diagnostic tools to detect biomarkers, metabolites, and drugs by extracting ISF, thus eliminating the need for complex, invasive, and repetitive blood sampling via subcutaneous injection [136]. To accurately monitor blood glucose levels, diabetic patients typically have to perform frequent fingertip tests to draw fresh blood, which is particularly painful and prone to the risk of cross-contamination. Ju et al. reported a novel SERS sensor based on low-cost PMMA MN arrays for in situ skin glucose detection. This polymer MN array-based SERS biosensor is minimally invasive to the skin, with no significant adverse reactions, and has the potential to be used for painless blood glucose monitoring in diabetic patients in the future (Figure 7a) [75]. Mei et al. explored the possibility of utilizing cutaneous ISF as a new way to diagnose acute peritonitis by enabling the patch sensing of H_2_O_2_ biomarkers for the dynamic tracking of acute peritonitis via SERS MN technology (Figure 7b) [35]. Xiao et al. utilized a microfluidic-based plasma MN biosensor for the ultrasensitive SERS monitoring of uric acid to enable the diagnosis of renal disease [113].

The deep tissue sensing via SERS MN-based biosensors can be a useful tool for the diagnosis of cancer, as such devices can be inserted directly into the tumor microenvironment or at the site of infection [118,137]. For example, TYR, the enzyme involved in melanin production by melanocytes, is overexpressed by malignant skin cells. As a result, this enzyme is usually enriched in skin ISF during cancer development [138]. He et al. constructed a SERS MN for the in situ detection of superoxide anion radicals (O_2_^·−^) for the early diagnosis of melanoma (Figure 7c) [36]. Huang et al. reported a SERS MN sensing platform that combines a wearable MN patch and trimetallic Au@Ag-Pt NPs for SERS and the colorimetric bimodal detection of in situ TYR in human skin for potential melanoma screening (Figure 7d) [80]. Gu et al. integrated a MN biosensor with SERS technology for TYR in situ detection in human skin using dopamine-functionalized AuNPs as a capture substrate and 4-MPBA-modified AgNPs as a SERS probe for the rapid and sensitive detection of screened early-stage melanoma [76]. Chen et al. combined MNs with a nano-Ag/MBL membrane (which has high photocatalytic and antibacterial properties) to provide a unique method for in situ breast cancer detection by extracting carcinoembryonic antigens (CEAs) from skin ISF [139]. Al Sulaiman et al. first reported the use of hydrogel MN patches coated with an alginate–peptide nucleic acid hybrid material to sample, extract, and detect specific miRNA biomarkers from skin ISF with faster kinetics. Furthermore, the development of MN-based biosensors for continuous cancer monitoring has recently attracted significant interest [140]. Once inserted into the dermal area, MNs can form microchannels that allow the loaded genes, biomolecules, or drugs to effectively bypass the stratum corneum (SC) and reach the tumor site, making MNs appear to be the safest alternative to oral or parenteral drug delivery [141,142]. In a study by Fu et al., a chemo-photodynamic therapy targeting breast cancer was developed by incorporating the chemotherapy agent cisplatin (CDDP) and IR 820 into a polyvinylpyrrolidone–vinyl acetate (PVPVA) copolymer as the core material for the MN patch. This combination of CDDP and IR 820 induced cell death, caspase-3 activation, and intracellular ROS generation. The results showed a significant inhibition of tumor development in terms of increased cell death and decreased cell proliferation [143]. MNs are used as a minimally invasive diagnostic tool for the early diagnosis of diseases, thus avoiding the pain of frequent blood collection for patients. Despite these advancements, challenges remain, including optimizing the stability and reproducibility of SERS substrates, enhancing the sensitivity for low-concentration analytes, and ensuring biocompatibility for clinical use. Overall, the integration of MNs and SERS represents a cutting-edge approach in analytical detection, with the potential to revolutionize point-of-care testing and personalized medicine.

### 5.2. Drug Monitoring

**Figure 8:**
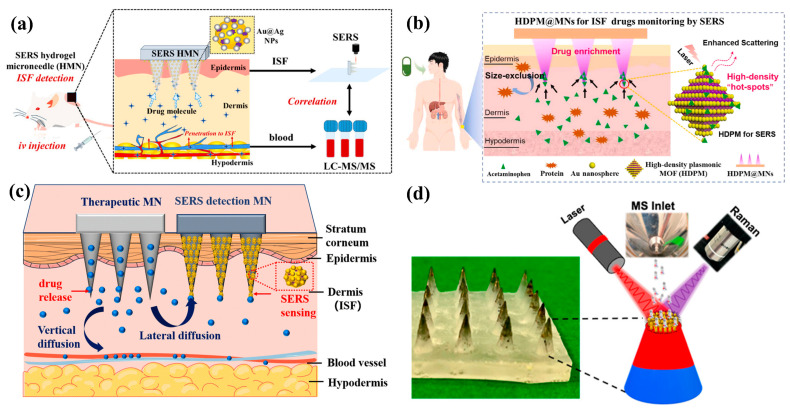
(**a**) Schematic diagram of SERS HMNs for drug concentration detection in dermal ISF. Reprinted with permission from [72]. Copyright 2024 American Chemical Society. (**b**) Schematic diagram of the principle for monitoring acetaminophen drug concentration in skin ISF with the plasmonic HDPM@MN SERS sensor. Reprinted with permission from [37]. Copyright 2024 Elsevier B.V. (**c**) Schematic diagram of the study of drug release and diffusion behavior of a therapeutic MN via a SERS MN in dermal ISF. Reprinted with permission from [144]. Copyright 2023 Royal Society of Chemistry. (**d**) Schematic diagram of SERS and mass spectrometry substrate MNs for the detection of synthetic drugs in plasma. Reprinted with permission from [38]. Copyright 2023 American Chemical Society.

Conventional drug monitoring requires the collection of large amounts of serum and labor-intensive sample preparation and analysis, which may take hours or even days to obtain results to guide patient treatment. The MN-based SERS analysis of ISF device is an ideal tool for drug monitoring. For example, Li et al. used methylene blue (MB) and mitoxantrone (MTO) as model drugs, employing a novel core–shell structure of an Au@Ag SERS substrate and hydrogel encapsulated on MNs. They achieved sensitive and quantitative drug monitoring in mouse ISF within 10 min. Their work proposed an effective ISF drug monitoring tool. Moreover, experiments demonstrated that the permeability of blood to ISF is drug-dependent, providing insightful information on the potential of ISF as an alternative to blood for in vivo drug detection (Figure 8a) [72]. Li et al. also designed a high-density plasma MOF (HDPM) SERS MN sensor for the real-time, non-destructive monitoring of acetaminophen (APAP) levels in ISF. The sensor showed a strong capability for APAP enrichment and monitoring, exhibiting excellent stability and reproducibility, with a lower detection limit of 0.45 μM. Additionally, by monitoring APAP concentrations in rat skin interstitial fluid at different doses and administration times, the HDPM@MN can be used to determine the pharmacokinetics of APAP in rats and the physiological features related to various dosing regimens. This work not only offers hope for drug monitoring but also provides a new approach for the non-destructive monitoring of other important low-abundance physiological biomarkers (Figure 8b) [37]. Kolluru et al. developed SERS MNs for collecting biomarkers in ISF. MNs create micropores in the skin surface, through which small amounts of ISF are collected onto the plasma paper on the back of the patch. The plasma paper is prepared by fixing AuNR coated with poly (sodium styrene sulfonate) onto a thin filter paper strip. This method was used to simulate the determination of drug pharmacokinetic characteristics in rat ISF and serum [29]. Shi et al. developed SERS MNs for the sensitive analysis of model drugs in ISF and used the SERS MN system to study the release and diffusion behavior of drugs in the ISF of live mice. Their work demonstrated that the application of skin heating or cupping treatment enhanced drug diffusion in the ISF, providing a new tool for the in situ and real-time detection of drug molecules in ISF. This will be beneficial for the development and evaluation of MN-based therapeutic systems (Figure 8c) [144]. Vitoria et al. reported the fabrication of nanoparticle-modified MNs, which is a sample ionization platform for SERS MN and substrate-supported electrospray ionization (ssESI) mass spectrometry (MS). By controlling various interfacial interactions, polymeric ligand-functionalized AuNRs were adsorbed onto superhydrophobic surface-modified PDMS MNs. Using this AuNR-decorated MN substrate, the synthetic drugs fentanyl and alprazolam were analyzed, with detection limits in the sub-picomolar range. A further analysis of drug–molecule interactions on the AuNRs surface using the Langmuir adsorption model revealed that fentanyl was more polarized and therefore could interact more strongly with the hydrophilic polymer layer on the surface of AuNRs. This method was further validated for detecting these two potent drugs in plasma samples from 10 drug overdose (DOA) patients based on SERS and ssESI-MS. A chemometric analysis of the SERS-based detection showed excellent classification between fentanyl, alprazolam, and their mixtures in the selected 10 samples. Most importantly, SERS MN analysis also successfully identified fentanyl and alprazolam in the same 10 DOA plasma samples. The multimodal drug detection approach proposed in this study is a highly versatile detection technique that can be applied to detect any type of drug without requiring any complex sample preparation (Figure 8d) [38].

### 5.3. Food Safety Analysis

**Figure 9:**
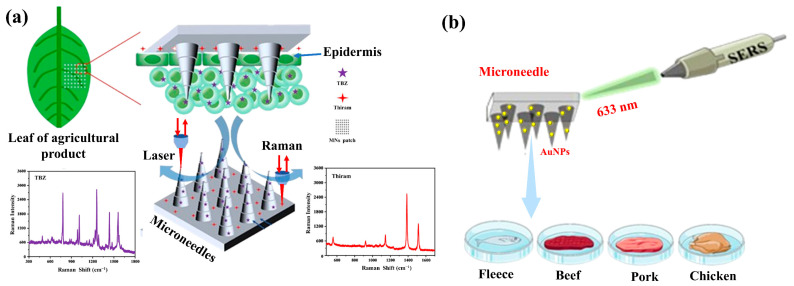
(**a**) Schematic diagram of the simultaneous detection of pesticide residues on the surface and inside agricultural products using a microneedle patch. The needles of the microneedle patch can detect the pesticide residues inside agricultural products by piercing the cuticles, and the basement of the microneedle patch can detect the pesticide residues on the surface by contact with the agricultural products; the leaf represents the agricultural product. Reprinted with permission from [5]. Copyright 2023 American Chemical Society. (**b**) Schematic diagram of the use of SERS microneedles for the detection of putrescine and cadaverine in meat samples. Reprinted with permission from [131]. Copyright 2025 American Chemical Society.

Pesticide residues are commonly found in various agricultural products such as tea, fruits, and vegetables. Some pesticide residues, especially endogenous pesticides, are difficult to remove and may cause diseases such as cancer, hormone disruption, asthma, allergies, and hypersensitivity reactions [145]. The issue of meat freshness is also common in fish, shrimp, beef, lamb, and chicken. The food safety problems caused by both have become some of the most widely discussed issues globally [146]. Therefore, developing effective MN-based SERS sensors for the collection and detection of pesticide residues and meat food safety factors is crucial. Yi et al. presented a new MN patch-based SERS sensor. The upper patch and lower needle of the MN could simultaneously detect pesticide residues on the surface and inside of agricultural products. The stepped structure of the MN increased the surface area of the sensor. The AgNPs and sodium hyaluronate/polyvinyl alcohol (HA/PVA) hydrogel used in this sensor effectively amplified the Raman signal of pesticide residues. The sensor could simultaneously detect thiram and thiabendazole pesticide residues with detection limits of 10^−7^ and 10^−8^ M, respectively, and the detection process was minimally invasive and harmless to the agricultural products (Figure 9a) [5]. Li et al. developed a MN-based SERS integrated with both redox-sensitive and pH-sensitive components to detect the real values of the redox state and the pH of fruits and vegetables. The SERS MN is inserted into the produce and then withdrawn to obtain the SERS signal, which indicates the pH and redox state of the fruit or vegetable. Using the MN-based SERS, the authors conducted tests on 10 types of fruits and vegetables, including different parts and depths of banana peel and pulp, as well as various parts of strawberries. The MN-based SERS was expected to serve as a multifunctional platform for the comprehensive assessment of fruits and vegetables in daily life, as well as in food processing and management [147]. Yang et al. developed a AuNPs@PDA@PMMA-MN SERS sensor. The AuNPs@PDA@PMMA-MN showed an enhancement factor of up to 1.74 × 10^6^ for R6G. Based on this, 4-MBA was modified onto the AuNPs@PDA@PMMA-MN substrate, successfully constructing a MN sensor for the selective detection of putrescine and cadaverine (Figure 9b). This sensor could quantitatively detect putrescine and cadaverine in meat, with detection limits of 2.43 × 10^−7^ mol/L and 9.93 × 10^−8^ mol/L, respectively. The method was successfully applied for the in situ detection of putrescine and cadaverine in real meat samples (fish, shrimp, beef, pork, and chicken). The AuNPs@PDA@PMMA-MN SERS sensor was proven to be simple to fabricate, highly sensitive, highly specific, and capable of online, in situ detection [131]. It is expected to have broader applications in food quality testing, serving as a reliable potential tool for assessing meat freshness and ensuring consumer health. In conclusion, the developed MN-based SERS sensor has great potential for applications in pesticide residues, agricultural products, and meat freshness monitoring.

### 5.4. Other Application

SERS MNs, due to their portability, speed, sensitivity, and minimally invasive nature, have also been applied in physiological health assessment and cosmetics analysis, providing innovative technological solutions for personalized care and health management. Critical to effective treatment and management in fields such as cosmetics and physical health assessment is continuous and accurate data monitoring. Han et al. prepared metal MNs with SERS activity for in vivo sampling and an ex vivo SERS assay of NO [28]. Park et al. demonstrated that integrating plasma-enhanced gold nanorods with a polymer MN array effectively monitors the pH of skin ISF. Plasma nanoparticles coated with pH-sensitive 4-MBA can be used to monitor pH levels in various samples. 4-MBA is protonated at an acidic pH (pH = 2) and deprotonated at a basic pH (pH = 12) [122]. The invention of flexible plasma substrates has paved the way for integrating SERS-based detection with MN arrays and wearable devices. To simultaneously detect redox potential and pH in rat joints, Pan et al. designed SERS MNs with two separate grooves, each containing redox-sensitive and pH-sensitive MNs. The composite SERS MNs were inserted into muscles with minimal invasiveness, enabling the detection of redox status and pH within 5 min, as well as monitoring the dynamic evolution of redox status and pH in the muscles. The multiplex SERS MNs were also inserted into rat joints, which lack flowing fluid, to detect their redox status and pH. The strategy of integrating multiple MNs into a single groove makes SERS MNs a versatile analytical tool for minimally invasive in vivo sampling and direct ex vivo Raman detection. Furthermore, these multifunctional SERS MNs are expected to become a powerful analytical tool for advancing biomedical research [127]. Chia et al. developed 3D SERS MNs with self-assembled AuNPs on PLA MNs using tannic acid (as a chemical adhesive and reducing agent) for in-depth chemical and biomolecular analysis. The LOD for small molecules was below 200 ppb, and the LOD for bacteria was below 10^2^ CFU cm^−2^. Au-MNs were used for photodynamic therapy, with the SERS monitoring of photosensitizer degradation. By applying 650 nm backside excitation to the Au-MNs on an agar plate (simulating a semi-aqueous agar skin model), 98.5% bacterial inhibition was achieved after photodynamic therapy [70]. Chen et al. developed a flexible MN-based SERS sensor for the convenient and sensitive pH monitoring of skin ISF. A flexible MN array was fabricated using NOA 65, and pH-sensitive 4-MBA-labeled AgNPs were electrostatically assembled onto the surface of the flexible MN array. The proposed flexible MN-based SERS sensor exhibited a good linear response in the pH range of 5 to 9, covering the normal pH levels of skin ISF. Using ISF extracted from pig skin as an experimental model, the pH values detected by the MN-based SERS sensor were in good agreement with the theoretical results. Furthermore, the proposed flexible SERS sensor demonstrated excellent mechanical strength, sensing stability, and biocompatibility both before and after skin insertion, making it a potential bioanalytical tool for practical pH level detection in ISF and providing technical support for SERS-based wearable biosensors [34]. In recent years, MN has attracted significant attention in cosmetic applications. Typically, MN-based beauty treatments have aimed to promote the natural healing of injured skin; address various skin conditions such as scar tissue, acne, facial rejuvenation, abnormal pigmentation, and hair loss; and improve skin permeability, while providing the effective delivery of active cosmetic molecules into the skin [148,149]. In conclusion, MN-based SERS biosensor technology will help to promote personalization in various fields.

## 6. Challenges of Translating from Lab to Clinical and Market Levels

The transition of MN-SERS systems from the laboratory to clinical application faces multidimensional challenges, with key limitations spanning technical hurdles, biocompatibility, clinical validation, and industrialization. The following key considerations need to be addressed: (1) The core limitations at the technical level mainly include the preparation accuracy and consistency of MN arrays, the in vivo interference and calibration challenges of SERS signals, and the specificity of multi-analyte detection and multi-plexing capability. (2) In situ SERS analysis by modifying metal materials on the surface of MNs is simple, but this subcutaneous insertion may cause toxicity or sample contamination. The heterogeneity caused by insufficient nanoscale control during the synthesis and fabrication of metal nanostructures also represents a core challenge restricting SERS signal reproducibility. Therefore, these limitations of SERS MN sensors need further improvement. (3) The accuracy of biological analysis when collecting ISF through MNs is uncertain, and the lack of clinical data on the correlation of biomarker concentrations between ISF and blood hinders the application of effective diagnostics. (4) The complex materials and processes of MN-SERS systems are expected to incur higher single-test costs, thereby posing challenges for widespread adoption in primary healthcare institutions. (5) Regulatory approval of the MN-SERS monitoring devices produced may also be a hurdle because this is a lengthy and complex process that requires rigorous preclinical and clinical testing [33]. Overall, preclinical validation experiments have demonstrated promising ISF collection and biomarker detection. Although some clinical trials are ongoing or their status remains unknown, their inclusion highlights the importance of exploring and validating MN-based SERS technologies for continuous health monitoring. However, we anticipate that upon the completion of clinical trials, some products will move closer to market level.

Translating laboratory-based MN-SERS monitoring research into marketable products presents several barriers. Firstly, scaling manufacturing processes to produce large quantities of MNs with consistent quality remains a key challenge. This endeavor requires investment in cost-effective scalable technologies (e.g., roll-to-roll manufacturing), which may prove difficult because materials used in scalable processes often differ from those at the research level. Such disparities necessitate the identification of alternative biocompatible materials and multiple iterative optimizations of devices for scalable fabrication. In addition, quality management practices and compliance with relevant standards are required to ensure the safety, reliability, and effectiveness of the devices. Finally, regulatory approval of the MN-SERS monitoring devices produced may also be a hurdle because this is a lengthy and complex process that requires rigorous preclinical and clinical testing [33]. In conclusion, with the development of wearable technology and the demand in point-of-care (POC) markets, MN-SERS monitoring devices hold the promise of revolutionizing health diagnostics, providing rapid and minimally invasive testing beyond traditional laboratory confines.

## 7. Conclusions and Perspectives

The development of SERS MN sensors has fundamentally transformed blood-centered invasive diagnostics by providing direct and continuous biological sample collection. Although MNs are a very effective sampling technology, they still face significant challenges and interdisciplinary barriers when entering the current market. This review comprehensively summarizes the latest advancements in MNs in SERS analysis to promote their personalized applications in the SERS field. SERS MN sensors made from metals, inorganic materials, and polymers are introduced, carefully crafted using advanced materials and bioengineering techniques to achieve continuous, accurate, reliable, and efficient diagnostics. Recent applications of MN-based SERS analysis in disease diagnosis, drug detection, and food safety are highlighted. Although many innovative works have been completed, MNs in SERS analysis still face many limitations that require continuous efforts.

Despite the multitude of challenges confronting the design of MN-based SERS sensors, the continuous research on SERS MN sensing systems offers infinite possibilities for analytical applications in fields such as biomedicine, disease diagnosis, and food safety. Firstly, to achieve consistent and accurate ISF collection using MNs under diverse skin conditions, improvements are required in MN design, material optimization, sampling protocols, and auxiliary technologies. The SERS MN system should also integrate bionic technology to better meet biomedical requirements. Secondly, integrating SERS MN sensors with power supplies, wireless communication, data acquisition, and visualization components into a closed-loop system is expected to facilitate the clinical translation of wearable technology [150,151]. Moreover, investigating MN-modified materials with excellent biocompatibility and enrichment effects can enhance the application advantages of SERS MN sensing systems in biomedicine, disease diagnosis, and treatment, as well as food safety analysis. Finally, the integration of artificial intelligence (AI) with MN-SERS systems represents a cutting-edge direction in current bioanalysis and intelligent diagnostics. This interdisciplinary fusion can enhance detection performance across multiple levels, including signal processing (AI-Enabled Signal Acquisition and Preprocessing) [152], data analysis (AI Applications in Spectral Analysis and Data Interpretation) [153], and system optimization (AI Support for System Optimization and Clinical Decision-Making) [154]. The SERS MN sensing system should be further combined with artificial intelligence to develop more effective POC devices [155], paving the way for patient-centered healthcare and clinical wearable devices.

## Figures and Tables

**Table 1:** Application of microneedles in SERS analytical sensing.

Application	Microneedle	Material Modification	Detection Mode	Analyte	Linear Range	LOD	Ref.
Disease diagnosis	PMMA	AgNPs@PDA	in situ	pH	4.0–8.0	/	[71]
				Redox potential	417.0–599.8 mV	/	
				ROS	0–480 ng/mL	/	
	NOA 65	AuNRs	in situ	pH	2.0–12.0	/	[122]
	PMMA	Au-Ag	ex situ	Tyrosinase	0.05–200 U/mL	0.05 U/mL	[76]
	PMMA	AuNPs	in situ	H_2_O_2_	3–100 μmol/L	3 μmol/L	[35]
	NOA 65	AgNPs	ex situ	pH	5.0–9.0	/	[34]
	NOA 61	AuNPs	in situ	O_2_^·−^	0–480 ng/mL	/	[36]
	PMMA	AuNPs	in situ	Pyocyanin	1–10 μmol/L	3.58 μmol/L	[124]
	PDMS	AuNPs	in situ	Uric acid	0.01–1 μmol/L	0.51 μmol/L	[113]
	PMMA	AgNPs	in situ	Glucose	0–20 mmol/L	/	[75]
	NOA	Au@Ag-Pt NPs	ex situ	Tyrosinase	0.05–200 U/mL	0.01 U/mL	[80]
	Silicon	AgNPs	ex situ	Gaseous 4-ethylbenzaldehyde	/	10 ppb	[125]
	Ag	Au@Ag	ex situ	Glutathione	0.1–15 mmol/L	0.037 mmol/L	[126]
	Stainless-steel	Gold nanoshells	ex situ	pH	4.0–8.0	/	[127]
				Redox potential	/	/	
	Stainless-steel	Gold nanoshells	ex situ	Glucose	0.5–1.5 g/L	/	[128]
	TMA/CAA/HEMA	AuNPs	in situ	*Escherichia coli*	0–1.0 × 10^10^ CFU/mL	5.5 × 10^4^ CFU/mL	[129]
				*Staphylococcus aureus*	0–5.2 × 10^9^ CFU/mL	1.2 × 10^4^ CFU/mL	
				*Acinetobacter baumannii*	0–5.0 × 10^9^ CFU/mL	1.4 × 10^4^ CFU/mL	
				*Pseudomonas aeruginosa*	0–8.3 × 10^9^ CFU/mL	5.5 × 10^2^ CFU/mL	
Drug monitoring	PMMA	Au@Ag	ex situ	Methylene blue	0–1 μmol/L	193.8 nmol/L	[72]
				Mitoxantrone	0–70 μmol/L	2.9 nmol/L	
	HDPM/PU/SF	AuNPs@MOF	in situ	Acetaminophen	1–100 μmol/L	0.45 μmol/L	[37]
	PVDF	Au-Ag	in situ	Levofloxacin	0–8 mmol/L	0.45 mmol/L	[130]
	PDMS	AuNRs	ex situ	Rhodamine 6G	0.05–100 μmol/L	/	[38]
	Plasmonic paper	AuNRs	ex situ	Fentanyl	10 fmol/L–1.0 μmo/L	68.5 fmol/L	[29]
				Alprazolam	1.0 pmol/L–100 μmol/L	5.6 pmol/L	
Food safety analysis	TMA/CAA/HEMA	Gold nanopopcorns	ex situ	*Escherichia coli*	2.4 × 10^2^–2.4 × 10^6^ CFU/g	143 CFU/g	[69]
				Putrescine	10^−7^–10^−3^ mol/L	2.43 × 10^−7^ mol/L	
	PMMA	AuNPs@PDA	in situ	Cadaverine	10^−7^–10^−3^ mol/L	9.93 × 10^−8^ mol/L	[131]
	HA/PVA	AgNPs	ex situ	Thiram	0–10^−7^ mol/L	10^−7^ mol/L	[5]
				Thiabendazole	0–10^−8^ mol/L	10^−8^ mol/L	
	Carbon fiber	Dendrite-like gold	ex situ	Acetamiprid	0.1–10 μg/mL	0.05 μg/mL	[56]
	Ag	AuNPs	ex situ	Malachite green	10^−8^–10^−5^ mol/L	0.1 nmol/L	[132]
Other analysis	PLA	AuNPs	in situ	*S. aureus*	10^2^–10^8^ CFU/cm^2^	100 CFU/cm^2^	[70]
			in situ	Purines	/	200 ppb	
	PLGA	Gold nanoflower	ex situ	Methylene blue	0.05–1.0 μmol/L	1.0 nmol/L	[68]
	NOA 63	AuNR	ex situ	Microplastics	/	0.5 μg/mL	[133]
	Stainless-steel	Gold nanoshells	ex situ	Lipopolysaccharide	/	/	[134]
	Au	Au thin film	in situ	Benzenethiol	10^−8^–10^−2^ mol/L	10^−8^ mol/L	[135]
